# Nanotechnology applications in hematological malignancies (Review)

**DOI:** 10.3892/or.2015.4100

**Published:** 2015-07-02

**Authors:** AHMED SAMIR, BASMA M ELGAMAL, HALA GABR, HATEM E SABAAWY

**Affiliations:** 1 Department of Clinical Pathology, National Cancer Institute, Cairo University, Cairo, Egypt; 2 Department of Clinical Pathology, Kasr Al-Ainy Faculty of Medicine, Cairo University, Cairo, Egypt; 3 Department of Medicine, RBHS-Robert Wood Johnson Medical School, Rutgers University, New Brunswick, NJ 08903, USA; 4 Rutgers Cancer Institute of New Jersey, New Brunswick, NJ 08903, USA

**Keywords:** nanotechnology, hematological malignancies

## Abstract

A major limitation to current cancer therapies is the development of therapy-related side-effects and dose limiting complications. Moreover, a better understanding of the biology of cancer cells and the mechanisms of resistance to therapy is rapidly developing. The translation of advanced knowledge and discoveries achieved at the molecular level must be supported by advanced diagnostic, therapeutic and delivery technologies to translate these discoveries into useful tools that are essential in achieving progress in the war against cancer. Nanotechnology can play an essential role in this aspect providing a transforming technology that can translate the basic and clinical findings into novel diagnostic, therapeutic and preventive tools useful in different types of cancer. Hematological malignancies represent a specific class of cancer, which attracts special attention in the applications of nanotechnology for cancer diagnosis and treatment. The aim of the present review is to elucidate the emerging applications of nanotechnology in cancer management and describe the potentials of nanotechnology in changing the key fundamental aspects of hematological malignancy diagnosis, treatment and follow-up.

## 1. Introduction to nanotechnology

Nanotechnology is the management of material properties at nanometer levels. The concept of nanotechnology was first introduced by the American physicist Richard Feynman who gave a talk entitled 'There's Plenty of Room at the Bottom' at an American Physical Society meeting at Caltech in 1959. Feynman did not mention 'nanotechnology', yet considered the potential to manipulate individual atoms at the molecular level. This talk inspired the conceptual beginning for nanotechnology science through creating the concept of 'molecular manufacturing' (1). The prefix 'nano' derives from the Greek word 'nanos' which means 'a dwarf'. A nanometer (nm) measures one-billionth of a meter. Many biological structures are within the nanometer size. While a single RBC is 2,500 nm, a DNA molecule is 2.5 nm, and while a protein molecule is 1–20 nm, the biggest amino acid (tryptophan) is 1.2 nm (2).

Nanotechnology is defined as the creation and use of structures, materials, devices and systems with completely novel properties and functions obtained from their size which ranges from 1 to 100 nm. It is applied in many fields including electronics, information technology, material developments and biomedicine (3). Nanobiotechnology represents a specific category of nanotechnology, based on biological molecules and processes that involve the creation of devices and systems at the nanolevel to examine or control biological processes (4,5).

Cancer nanotechnology is a striving field (6), current methods for cancer diagnosis have limitations regarding the sensitivity and the ability to detect cancer at early stages (7). In addition, conventional treatment options have considerable side-effects due to non-target tissue toxicity and their success is limited in advanced disease stages due to the development of drug resistance. An additional diagnostic and follow-up problem is related to the detection of minimal residual diseases (MRD) after treatment (7). Therefore, there is an urgent need to develop novel therapies and cell type-specific delivery systems to deliver anticancer therapies more effectively. Hematological malignancies representing cancers that affect blood, bone marrow and lymph nodes, are associated with these same problems encountered with other types of cancer. Nanotechnology may find novel solutions to all of these problems. New nanodevices can perform different key functions such as detecting cancer cells at the initial stages and determining their location in the body. Nanotechnology, either alone or in combination with traditional diagnostic methods, provides new sensitive, specific, reproducible and cost-effective methods for diagnosis of different types of cancers including hematological malignancies. In the field of cancer therapy, nanodevices are used to carry drugs to specific target cells and tumor sites, with nanoparticle-based drug delivery approaches making remarkable success in site-specific release of different therapies due to their unique physical, chemical and biological features.

The advantages that nanotechnology offers include targeted delivery of drugs, reduced dosage, reduced frequency of dosing, better solubility, reduced immunogenicity and better half-life of these drugs when used *in vivo*. Multiple types of nanomaterials can be used to generate the nanoscale drug delivery systems including lipid-based nanocapsules or liposomes and natural or synthetic polymers. Liposomes are biocompatible vesicles formed of amphipathic lipid bilayer membranes. Polymers could be derived from biocompatible drug conjugates or micelles that self-assemble to form a hydrophilic outer corona and a hydrophobic inner core for drug encapsulation. The choices of different nanoscale systems are based on the components, applications, advantages and limitations of each preparation. These novel nanotherapeutics are rapidly progressing as they offer solutions to many limitations of conventional drug delivery systems such as nonspecific targeting, limited biodistribution, poor bioavailability and low therapeutic indices. Factors that determine nanomaterial design and characterization include size and shape, blood half-life, controlled drug release and active targeting of nanoparticles. Efficient cell uptakes were reported with nanoparticles with a size range of 40–50 nm (8). Studies examining the effects of nanoparticle shapes demonstrated that rod-shaped micelles continue in the circulation 10 times longer than spherical micelles (9), while these spherical micelles bind more tightly to circulating and leukemic cells (10). Moreover, nanoscale formulations can be rendered multifunctional with the concept of combination therapy in practice. In the following section, a number of applications of nanotechnology in hematological malignancies will be discussed with a particular emphasis on the utilization of nanotechnology in diagnosis, treatment and follow-up of different types of leukemias and lymphomas.

## 2. Nanotechnology applications in different types of leukemia

### Nanotechnology applications in acute leukemias

Acute leukemias represent a group of cancers caused by malignant transformation of hematopoietic cells and usually associated with maintaining a partial capacity of differentiation of the progenitor tumor initiating cells (11). In general, acute leukemias are divided into acute myeloid (also called non-lymphocytic) leukemia (AML) (12) and acute lymphoid leukemia (ALL) (lymphoblastic leukemia/lymphoma) (13). ALL has a higher incidence in children, while AML usually predominates in adults, yet age profiles overlap. Sex distribution for AML is almost equal until the age of 60, after which a considerable male predominance occurs. The following section will discuss the different diagnostic and therapeutic applications of nanotechnology in acute leukemia.

### Aptamer-conjugated nanoparticles for selective detection of leukemic cells

Diagnosis and classification of acute leukemias depend on integration of information from morphologic and cytochemical examination along with data obtained from immunophenotyping analysis using flow cytometry (FCM) and cytogenetic or molecular analyses of diagnostic genetic mutations associated with prognosis (14). As antibodies used in FCM may not detect all the molecular events associated with the development of malignancy or other factors that may be useful for clinical management, the study of cell surface proteins with nanotechnology may improve treatment strategies. Creating probes to detect surface proteins can be used to classify tumors depending on the molecular features of these cells rather than their tissues of origin (15). In this regard, using molecular aptamers is a new strategy of identifying novel biomarkers that can be used to increase effectiveness of therapy and survival of leukemic patients (16). Aptamers are synthetic nucleic acid ligands that can be produced against many targets including proteins, drugs and amino acids (17). Compared with antibodies, aptamers have several advantages. To name a few, aptamers have a higher affinity and are easily synthesized with limited toxicity. In addition, aptamers can also fold into three-dimensional conformations with unique ability to bind biomolecular ligands (18). Making use of these advantages, Herr *et al* (19) have chosen an aptamer for acute leukemic cells with a specific sequence. In this study, they created an assay using aptamer-conjugated nanoparticles for quick recognition of acute leukemic cells using high-affinity DNA aptamers for signal detection. An oligonucleotide of 88 base pairs, which has the capability of binding to acute leukemic cells was attached to magnetic and fluorescent nanoparticles. The fluorescent nanoparticles then provided a method of detection, while the use of magnetic nanoparticles eliminated the need for centrifugation and helped the removal of unwanted particles.

### Enhanced leukemic cell detection using a novel magnetic needle and nanoparticles

Detection of MRD in leukemia is essential for follow-up of patients in order to change treatment strategies. Although the utilization of morphological examination of blood and bone marrow for detection of MRD has decreased, being replaced by more sensitive techniques, it is still being used as one of the easiest methods for detection of MRD (20). Morphological examination of peripheral blood and bone marrow has been used for years for detection of MRD. However, microscopic diagnosis is subject to the examiner's interpretations and depends on the experience of the hematologist, therefore distinguishing leukemic cells from normal lympho-hematopoietic progenitors can be challenging (21). Traditional methods for detection of MRD include FCM, polymerase chain reaction (PCR) as well as immunoglobulin (Ig) and T-cell receptor (TCR) gene rearrangements. These methods have a detection limit of 0.001% leukemic cells therefore decreasing dependence on morphological examination for detection of MRD (22). In order to improve the sensitivity of detection of leukemic cells in the bone marrow, Jaetao *et al* (23) developed a new device that uses antibodies coupled to superparamagnetic iron oxide nanoparticles (SPION) that were directed against the acute leukemia antigen CD34 linked with a magnetic needle biopsy. CD34-conjugated nanoparticles bound highly to CD34 expressing cell lines. In addition, the magnetic needle allowed the recognition of cells from cell lines and patient leukemic cells diluted into normal blood at low concentrations. They also observed that the magnetic needle improved the percentage of blasts visible by light microscopy by 10-fold indicating that using this needle may improve the detection levels for MRD (23).

### Therapeutic nanotechnology approaches in acute leukemia

After approval of Doxil, a pegylated liposomal doxorubicin by the US Food and Drug Administration (FDA) in 1995 for treatment of metastatic ovarian cancer and AIDS-related Kaposi sarcoma, several other drugs with similar features have been developed and were either approved or in different phases of clinical trials (24). Nanotechnology has a high potential for improving current therapeutic approaches in leukemia. In 2012, vincristine sulfate liposome injection (VSLI) was approved by FDA for treatment of adult patients with Philadelphia chromosome-negative ALL when those patients are in relapse or have progressive disease after two or more anti-leukemia therapies (25). Several other approaches have also been investigated. One of the approaches tested by Krishnan *et al* (26) was the incorporation of dexamethasone, which is commonly used for treatment of childhood leukemia, in polymeric NPs and testing the ability of the conjugate against leukemic cells *in vitro* and *in vivo*. The cytotoxic activity of the complex was similar to that of free dexamethasone. *In vivo* models have shown that the complex enhanced the quality of life of mice in comparison with dexamethasone alone and that NPs were cleared from the body with time, providing evidence for the potential use of this method to deliver dexamethasone in lower doses and more efficient forms. Another approach is incorporating different compounds into lipid nanoparticles. Lipid nanoparticles have many advantages as drug carriers being biocompatible, biodegradable, with a low toxicity and able to incorporate different drugs types. This approach was tested by Rahman *et al* (27) who loaded zerumbone, a natural dietary compound with an antitumor effect and a low water solubility, into nano-structured lipid carriers. The activity of the formulation was tested in human T-cell ALL cell lines and has shown a sustained release and a highly effective killing effect against these ALL cells.

Conjugation of drugs with colloids was also investigated. Cosco *et al* (28) investigated the effect of linking cytarabine (Ara-C) with one product of squalene which is a natural product used as a precursor in the synthesis of sterols. The product was assessed against different cancer cell lines (L1210, K562 and MCF-7) and has shown an effect against resistant leukemic cells (L1210R) compared with the naked compound as the conjugate protected the drug from cellular metabolic degradation. These results were also confirmed by an *in vivo* leukemic model in which mice treated with the conjugate had a better survival rate, than those treated with Ara-C alone.

### Nanotechnology for reversal of multidrug resistance (MDR) in leukemia

A major problem that represents an obstacle to the success of treatment of leukemia is the development of MDR, which is responsible for ~90% of cancer treatment failure (29). As MDR develops, cancer cells become resistant to the cytotoxic effects of chemotherapeutic agents (29). Using higher doses of these chemotherapies does not overcome the effects of MDR. On the contrary, it is frequently associated with several toxic effects creating a need for the development of a new approach combining conventional methods with new strategies to increase the delivery and concentration of drugs in target tissues (30). Numerous mechanisms have been implicated in the development of drug resistance in patients with AML. One of the common mechanisms is the development of efflux pumps, such as P-glycoprotein and MDR-associated protein (MRP) (31). An approach that may be used to overcome MDR is utilizing antibody drug conjugates (ADCs) by linking an antibody (or an antibody fragment) to a cytotoxic drug to improve the anticancer effect of antibodies and decrease the toxicity of the conjugated drugs (32).

Chen *et al* (33) prepared Fe_3_O_4_-MNPs (magnetic nanoparticles) loaded with adriamycin and tetrandrine to interfere with MDR of K562/A02 cells. Measurement of several biological parameters has shown that adriamycin- and tetrandrine-loaded Fe_3_O_4_-MNPs can help in counteracting the effects of MDR in K562/A02 cells, due to the buildup of drugs caused by its polymerization with Fe_3_O_4_-MNPs. In another study, Chen *et al* (34) synthesized tetraheptylammonium-capped MNPs-Fe_3_O_4_ causing an increase in the concentration of daunorubicin in MDR leukemia K562/A02 cells and augmenting the therapeutic effect of daunorubicin in MDR leukemic K562/A02 cells. This study also showed that utilizing daunorubicin only is less sensitive than daunorubicin associated with MNPs-Fe_3_O_4_. *In vitro* cytotoxicity assays provided evidence that MNPs-Fe_3_O_4_s are biocompatible materials. Combination of Fe_3_O_4_-MNP with other therapeutic forms can also represent an alternative strategy to overcome MDR in leukemia. Magnetic drug targeting (MDT) is a novel approach developed to increase the concentration of anticancer drugs in the tumor and decrease the amount of leakage into the surrounding healthy tissues (35). Ren *et al* (3) investigated this approach and combined Fe_3_O_4_-MNP with hyperthermia and chemotherapy in nude mice harboring tumor xenografts. Pathological examination of the tumors and markers of apoptosis were assessed. According to their results, tumors became smaller in size and apoptosis was observed in cells from the treated group, providing evidence for the benefits of using this approach to reverse MDR in leukemia.

Another promising approach to overcome MDR is the use of nanodiamonds (NDs). Several advantages of NDs favor their use in the field of medicine such as being inert, transparent, having high surface area and being biocompatible (36). NDs can be used as carriers for drugs or therapeutic nucleic acids. Man *et al* (37) adjusted different parameters such as pH and concentration for loading daunorubicin into NDs. The resultant complex was tested in K562 human leukemic cells, with MDR induced by gradual exposure to daunorubicin. The results showed that NDs could help the release of daunorubicin and overcome mechanisms of drug efflux, which induce resistance, therefore adding further evidence for the potential use of NDs to overcome MDR. In another study, Ghoneum *et al* (38) examined the effects of using a mixture of NDs and nanoplatinum (NP) known as DPV576 to overcome MDR in MDR human myeloid leukemic cells (HL60/AR) and MDR-sensitive cells (HL60). According to their results, the combination of daunorubicin with DPV576 was associated with higher incidence of the development of cell membrane holes in the MDR cells in comparison with the control cells that allowed better delivery of the drugs inside such cells.

### Modifications of all-trans retinoic acid for treatment of acute promyelocytic leukemia

Acute promyelocytic leukemia (APL) is a specific subtype of AML. The hallmark of APL is the presence of specific t(15;17) chromosomal translocation. The translocation occurs between retinoic acid receptor-α (RARα) gene and the PML gene on chromosomes 17 and 15, respectively (39). APL cells are induced by all-*trans* retinoic acid (ATRA) to differentiate into mature myeloid cells and ATRA may induce complete remission in APL patients (40). However, several complications appear to be associated with the use of ATRA including hypertriglyceridermia, mucocutaneous dryness and headache. In addition, ATRA resistance may develop due to lower plasma concentration of the drug leading to recurrence of the disease. Kim *et al* (41) developed new nanoparticles based on creating a complex between low molecular weight water-soluble chitosan (LMWSC) and ATRA. LMWSC has many advantages, in addition to being water soluble, including ease of modification and potential use as a gene or drug carrier. In their study, Kim *et al* (41) observed that ATRA was released from the nanoparticles for 10 days and had superior effects on CT-26 colon carcinoma cells on day 1, but the same cytotoxicity on day 2. These results provide proof-of-concept of using LMWSC nanoparticles in the drug delivery field. However, overcoming treatment resistance in APL is still a challenge. Understanding the molecular mechanisms associated with resistance and development of targeted therapy could be the ultimate solution to this problem (42).

### Nanotechnology applications in chronic myeloid leukemia (CML)

Due to their unique properties, gold nanoparticles (AuNPs) are among the interesting materials for use in the field of nanotechnology. AuNPs have many optical properties that make them suitable for bioimaging applications (43). AuNPs have superior absorption and scattering properties than traditional organic dyes. The size and shape of AuNPs affect their optical properties where they can absorb light from visible to the near-infrared (NIR) region raising interest for their medicinal use (44). Light absorption, scattering and emission are enhanced in AuNPs due to the interaction of light with the free electrons in the particles. Accordingly, the field of light causes oscillation of the conduction band electrons at the surface of the particles. This oscillation of free electrons in resonance with the electro-magnetic field is called the surface plasmon resonance (SPR), which is responsible for the unique properties of AuNPs (43).

AuNPs have also proven to be biocompatible, have limited toxicity and low susceptibility to photobleaching making them suitable for use in studies with human cells (45). These exceptional optical properties together with easy synthesis and functionality of AuNPs with different biomolecules allow the use of AuNPs for molecular assays and permit the detection of very low concentrations of nucleic acids, therefore providing five times higher sensitivity than fluorescence based techniques (46). Using gold nanoparticles in molecular assays can improve current technique performances or help in the development of completely new assays, which can be faster, more sensitive and less expensive. This is of great importance particularly for use in developing countries (45,47).

CML is one of the examples in which AuNPs have been used for molecular diagnosis. CML is one of the most common myeloproliferative disorders. It is a clonal disorder of hematopoietic stem cells that passes through three stages. The first phase is the chronic phase characterized by a marked increase in the total leukocytic count with predominance of progenitors and mature cells of myeloid nature. With time, the disease progresses into an accelerated and finally a blastic phase characterized by maturation arrest concomitant with the appearance of an increased number of blasts (48). Most CML patients, ~90%, have an acquired translocation between the long arms of chromosomes 9 and 22 t(9;22 q34;q11) resulting in what is known as the Philadelphia chromosome (Ph). The translocation results in the formation of fusion gene between BCR and ABL genes with production of fusion protein that has a greater tyrosine kinase activity than normal. The new gene encodes p210 BCR-ABL1 protein, instead of the p145 Abelson protein causing growth factor independence and unlimited growth of leukemic cells (49). Common procedures for the diagnosis of Philadelphia chromosome include karyotype analysis, fluorescence *in-situ* hybridization (FISH) as well as reverse transcriptase polymerase chain reaction analyses (RT-PCR). However, those methods are costly and time-consuming creating the need for developing methods that are sensitive and cost effective at the same time (50).

**Figure 1:**
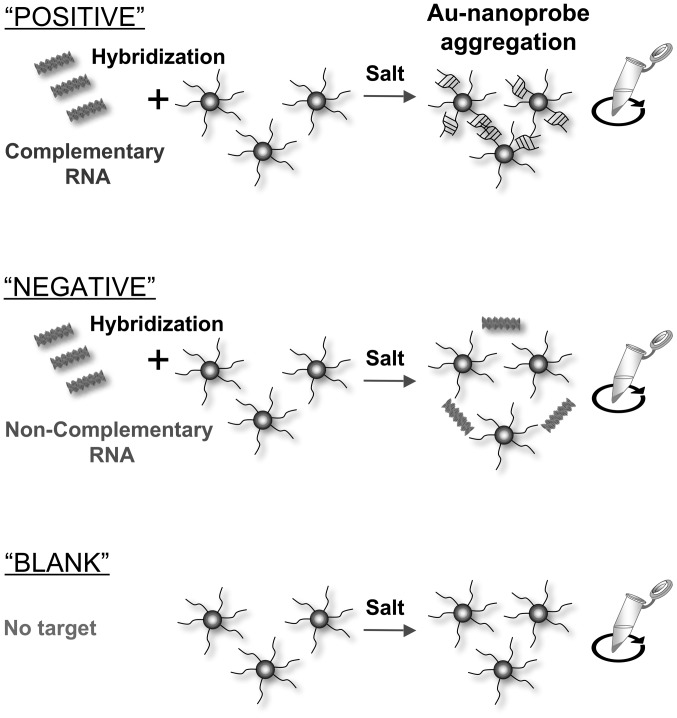
Schematic representation of the Au-nanoprobe assay method. The assay depends on the improved stability of the Au-nanoprobes when they hybridize with the complementary RNA in solution. Non-hybridized Au-nanoprobes aggregate upon the addition of salt. Positive, sample with existing RNA target; negative, sample with non-complementary RNA; blank, Au-nanoprobe alone (no target).

Conde *et al* (51) presented an Au-nanoparticle-based method for the diagnosis and quantification of the BCR-ABL fusion transcript associated with CML. The technique depends on the optical properties of AuNPs. According to the size, Au-nanoprobes absorb light in different regions of the spectrum. Au-nanoprobes of 13 nm are red and have a narrow SPR band ~520 nm. On the other hand, when aggregated Au-nanoprobes are present, the solution is blue. The colors of the solutions are compared before and after aggregation induced by salt either visually and/or using spectroscopy. As shown in Fig. 1, the existence of a complementary target does not allow aggregation and the solution appears red (SPR peak at ±520 nm), while the absence of this target allows aggregation of Au-nanoprobes, associated with alteration of color from red into blue (red-shift of the SPR peak to 600–650 nm).

The same team worked on another idea within the same scope. Baptista *et al* (52) have developed nanoparticles in the form of an alloy with different metal compositions and have linked them with different thiol-modified single stranded DNA (nanoprobes). This was used for simultaneous detection of multiple targets depending on the different colors detected by the different alloy composition. In this study, the targets detected were various gene products in CML; ABL, BCR and BCR-ABL fusion product allowing identification of multiple targets at the same time. The presence of BCR-ABL fusion gene transcript also provided the base for targeted therapy for CML. Imatinib mesylate (IM), a tyrosine kinase inhibitor which was introduced in 1998, acts selectively against the oncoprotein BCR-ABL with a high success rate (53). Despite major hematological and cytogenetic responses achieved by IM, resistance to IM develops in a significant number of patients, resulting in disease relapse (54). Palamà *et al* (55) developed new polyelectrolyte complexes to act as carriers for IM in KU812 CML cell line and CD34-positive cells collected from patients. The results of these studies have shown that the used complexes allowed longer BCR-ABL kinase inactivation even at lower doses of IM compared with the previously formed microscale formulation polyelectrolyte microcapsules, giving hope that such complexes can be used with success to overcome drug resistance and prevent relapse in CML patients (55). A major limitation to this approach is that failure of therapy in most CML cases is due to persistence of leukemic stem cells (LSCS) in which tyrosine kinase therapy (TKI) is not effective. A better understanding of the pathways regulating LSC survival and the mechanisms involved in disease progression is a major need to overcome resistance of therapy and relapse (56).

### Nanotechnology applications in B-chronic lymphocytic leukemia (B-CLL)

Chronic lymphocytic leukemia (CLL) is a clonal disorder characterized by accumulation of mature-appearing lymphocytes in the peripheral blood, bone marrow, lymph nodes as well as the spleen. CLL cells are frequently monoclonal B-lymphocytes that express CD19, CD5 and CD23, with weak or no expression of surface immunoglobulin (Ig), CD20, CD79b and FMC7 (57). CLL cells are also characterized by resistance to apoptosis. Although most CLL cells are non-replicating cells in G0 phase of the cell cycle, a small percentage of cells are replicating and these account for disease progression. The arrest of cells at this stage might explain the resistance of these CLL cells to cytotoxic drugs, which work through induction of apoptosis. Bcl-2 overexpression is one of the specific genes that is overexpressed in CLL and may induce such resistance (58).

The other finding that can affect growth and development of CLL is the presence of abnormal vascularization. CLL cells manufacture and release vascular endothelial growth factor (VEGF) and express VEGF membrane receptors (VEGF-R1 and VEGF-R2). VEGF also plays a significant role in CLL cells resistance to apoptosis (59). These findings can create the base for using targeted therapy with anti-VEGF antibodies (avastin; bevacizumab) to induce apoptosis in CLL cells. It has been shown that avastin selectively induces apoptosis in CLL cell through the mitochondrial pathway of caspase activation while sparing normal lymphocytes (60). However, a limitation to such an approach is the high concentration of the antibody necessary to achieve reasonable effects (58). Mukherjee *et al* (58) studied the functionality of gold nanoparticles with anti-VEGF antibodies on the release of the drug. In their study, all samples examined have shown a response to gold-AbVF that was considerably superior to CLL cells exposed to only AbVF or GNP with downregulation of anti apoptotic proteins. GNP alone has shown some response with induction of apoptosis in some of these CLL cells. Phase II clinical studies conducted on 46 CLL patients concluded that while anti-VEGF therapy, including avastin, remains a viable therapy for CLL, using a single agent anti-VEGF monotherapy had limited activity in CLL patients, and combination therapy is a more feasible approach particularly for patients with relapsed/refractory CLL (61). The combination of monoclonal antibodies, such as anti-CD20 or rituximab, with other types of chemotherapy is another promising approach for treatment in CLL (62).

Another strategy, developed later by Yu *et al* (63), recognized that anti-CD37 monoclonal antibody immunoliposomes could be used as carriers for specific targeting of B-CLL cells. Anti-CD19 or anti-CD20 was combined with anti-CD37 to form dual immunoliposomes that were used to induce apoptosis in B-CLL cells. Their results have proven that using the dual liposomes have higher effects regarding induction of apoptosis than using either anti-CD19 immunoliposome or anti-CD20 immunoliposome suggesting that this strategy can be favorable for personalized treatment of B-CLL and B-cell malignancies in general.

## 3. Nanotechnology applications in lymphomas

### Nanotechnology applications in Hodgkin lymphoma (HL)

CD30 is an antigen that is expressed on T-cells, activated B-cells as well as natural killer cells and has a very low expression on normal cells. Therefore, CD30 is an ideal target in classical HL. Brentuximab vedotin (BV) is a CD30-directed antibody-drug conjugate that received approval from FDA for the treatment of patients with HL after relapse (64). CD30 has also been investigated as a target for photothermal therapy using gold nanoparticles. Gold nanoparticles have several advantages that enhance their potential as important agents in nanotechnology. In addition to their absorption and scattering properties, gold nanoparticles can absorb light and switch it into heat. This property can be used to induce killing of cancer cells through protein denaturation and induction of apoptosis. Photothermal therapy also allows for monitoring of the process that eventually will lead to the death of the cancer cells (65). Accordingly, Zharov *et al* made use of this property for photothermal therapy of HL. They developed two gold nanoparticle-antibody conjugates, one of them was combined with an anti-CD30 receptor which binds to CD30 on the surface of L-428 Hodgkin cells and the other with an anti-CD25-receptor as a control. High killing power was achieved using appropriate doses of laser irradiation and gold concentration for gold-targeted L-428 cells with little to no effect on neighboring non-targeted cancer cells. These data further support previous findings for the potential use of gold nanoparticles as a safe modality for treatment of cancer.

### Nanotechnology applications in anaplastic large cell lymphoma (ALCL)

**Figure 2:**
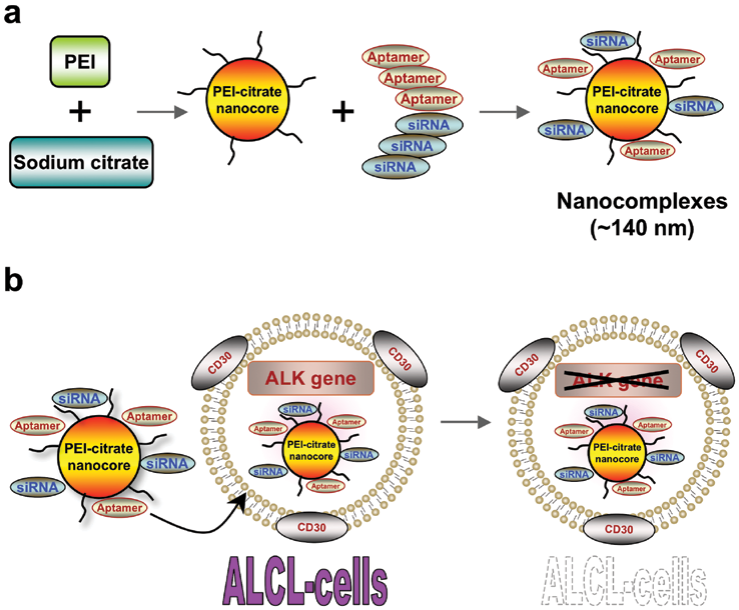
Development of a tumor cell-selective and cancer gene-specific nanocomplex for ALCL cells. (a) A nano-sized transporter structure is originally created as a nucleus through aggregation of polyethyleneimine (PEI) and crosslinking with sodium citrate (PEI-citrate nanocore). The synthetic RNA-based CD30 aptamers and ALK siRNA are then integrated onto the PEI-citrate nanocore to synthesize the nanocomplex. (b) When the RNA nanocomplex is added to the cultures, the aptamer part will specifically affect the CD30-positive ALCL cells. The aptamer-mediated cell binding will help the intracellular release of the nanocomplex. The siRNA part will then silence the cellular ALK gene, causing arrest in the growth of the ALCL cells. ALCL, anaplastic large cell lymphoma; ALK, anaplastic lymphoma kinase.

ALCL is a type of T-cell lymphoma with aggressive features and poor prognosis. One of the main features that characterize ALCL cells is abnormal expression of the anaplastic lymphoma kinase (ALK) together with the surface expression of CD30. Based on the presence or the absence of ALK translocation, the tumor is classified into either ALK-positive or -negative-ALCL. The presence of these unique and specific features can provide the base of targeted therapy for treatment of ALCL (66). For example, BV has been approved, in addition to its role in treatment of HL, for treatment of relapsed systemic ALCL (67). Crizotinib, an ALK inhibitor, is another FDA-approved drug for treatment of ALK-positive anaplastic large-cell lymphoma. However, resistance to crizotinib may develop limiting its use for long-term therapy (68). Using an approach that combines both characteristics, Zhao *et al* (69) have developed an RNA aptamer, which binds only to CD30 protein in solution. The complex produced was formed by inserting an RNA-based CD30 aptamer probe and ALK siRNA into polyethyleneimine-citrate carriers in nanosize (Fig. 2). According to their results, this complex allowed silencing of the expression of ALK gene in ALCL cells, stopping the growth of these cells with induction of apoptosis.

### Nanotechnology applications in mantle cell lymphoma

Mantle cell lymphoma (MCL) is a high-risk subtype of NHL characterized by an aggressive clinical course and by having the worst prognosis among B-cell NHL (70). These characters create a need for development of new agents and targets against this type of cancer. One of the agents that has attracted attention is lenalidomide, an immunomodulatory agent, that has been tested with some success for treatment of refractory MCL (71). Using high-throughput techniques can provide a better understanding of the mechanisms underlying excessive proliferation and apoptotic resistance in MCL, thus allowing the identification of new targets for therapy (72). One of these potential targets is SYK, which is one of the factors that control apoptosis. Overexpression of SYK is associated with several B-cell malignancies including MCL. Cely *et al* (73) designed a nanotechnology platform targeting specifically a SYK inhibitor in the MCL cells. According to their results, the developed liposomal nanoparticles (NP) loaded with a product called compound 61 (C-61) were able to induce apoptosis of the MCL cells in one day offering the base for using this therapeutic innovation against a large spectrum of lymphoid malignancies, including MCL.

## 4. Conclusions and future prospects

Nanotechnology represents a promising technology that can be of great value in the war against cancer. However, attention should be put towards safety issues and ethical concerns. Since particles at the nanosize have unique physical and chemical properties, care must be taken when dealing with particles or products for *in vivo* applications. Possible changes in the reactions of the particles with cells of the body may occur and changes in the absorption and secretion properties of such particles should be well investigated. FDA has recommended that meticulous research should be conducted in areas related to these novel properties that may cause toxicity. FDA also recommended that further research for development of devices and methods that can be used for measurement of nanoscale materials should be developed. Another important issue that is related to nanotechnology, being an emerging technology, is the ethical concern. In many cases, as science leaps forward, ethics lag behind. Part of the funding should be directed towards ethical, legal and social issues related to nanotechnology. One of the most famous examples for this readiness was during the Human Genome Project when James Watson suggested that 3–5% of the budget be devoted for study of ethical, legal and social issues.

The future prospects of nanotechnology and nanomedicine are very promising. Nanotechnology development for targeted therapy of cancer is one of the most interesting areas in research currently underway where drugs are delivered specifically to the diseased tissues or cells of interest without affecting nearby normal cells. Development of biomarkers for early detection and imaging of cancer cells is another important topic for research using nanotechnology. Scientists hope that progress in proteomics and bioinformatics can be used by nanotechnology to identify and kill malignant cells with the highest efficacy and the least possible side-effects, therefore bringing us closer to the goal of more effective cancer care.
